# BRAF^V600E^ mutation impinges on gut microbial markers defining novel biomarkers for serrated colorectal cancer effective therapies

**DOI:** 10.1186/s13046-020-01801-w

**Published:** 2020-12-14

**Authors:** Nadia Trivieri, Riccardo Pracella, Maria Grazia Cariglia, Concetta Panebianco, Paola Parrella, Alberto Visioli, Fabrizio Giani, Amata Amy Soriano, Chiara Barile, Giuseppe Canistro, Tiziana Pia Latiano, Lucia Dimitri, Francesca Bazzocchi, Dario Cassano, Angelo L. Vescovi, Valerio Pazienza, Elena Binda

**Affiliations:** 1 Cancer Stem Cells Unit, ISBReMIT, IRCSS Casa Sollievo della Sofferenza, Opera di San Pio da Pietrelcina, San Giovanni Rotondo, FG Italy; 2 Gastroenterology Unit, IRCSS Casa Sollievo della Sofferenza, Opera di San Pio da Pietrelcina, San Giovanni Rotondo, FG Italy; 3 Oncology Laboratory, IRCSS Casa Sollievo della Sofferenza, Opera di San Pio da Pietrelcina, San Giovanni Rotondo, FG Italy; 4 StemGen SpA, Milan, Italy; 5 Abdominal Surgery Unit, IRCCS Casa Sollievo della Sofferenza, San Giovanni Rotondo, FG, Italy; 6 Division of Medical Oncology, IRCCS Casa Sollievo della Sofferenza, San Giovanni Rotondo, FG Italy; 7 Anatomical Pathology Unit, IRCCS Casa Sollievo della Sofferenza, San Giovanni Rotondo, FG, Italy; 8 Science Directorate, IRCCS Casa Sollievo della Sofferenza, San Giovanni Rotondo, FG Italy; 9 Cancer Stem Cells Unit, Fondazione IRCCS Casa Sollievo della Sofferenza, Institute for Stem Cell Biology, Regenerative Medicine and Innovative Therapeutics (ISBReMIT), 71013 San Giovanni Rotondo, FG Italy

**Keywords:** Serrated human *BRAF*^V600E^ colorectal carcinoma (CRC), Gut microbiota, CRC biology and biomarkers, *BRAF*^V600E^ CRC non-invasive diagnosis, Anti-*BRAF*^V600E^ CRC patient-tailored strategies

## Abstract

**Background:**

Colorectal cancer (CRC) harboring *BRAF*^V600E^ mutation exhibits low response to conventional therapy and poorest prognosis. Due to the emerging correlation between gut microbiota and CRC carcinogenesis, we investigated in serrated *BRAF*^V600E^ cases the existence of a peculiar fecal microbial fingerprint and specific bacterial markers, which might represent a tool for the development of more effective clinical strategies.

**Methods:**

By injecting human CRC stem-like cells isolated from *BRAF*^V600E^ patients in immunocompromised mice, we described a new xenogeneic model of this subtype of CRC. By performing bacterial 16S rRNA sequencing, the fecal microbiota profile was then investigated either in CRC-carrying mice or in a cohort of human CRC subjects. The microbial communities’ functional profile was also predicted. Data were compared with Mann-Whitney U, Welch’s t-test for unequal variances and Kruskal-Wallis test with Benjamini–Hochberg false discovery rate (FDR) correction, extracted as potential *BRAF* class biomarkers and selected as model features. The obtained mean test prediction scores were subjected to Receiver Operating characteristic (ROC) analysis. To discriminate the *BRAF* status, a Random Forest classifier (RF) was employed.

**Results:**

A specific microbial signature distinctive for *BRAF* status emerged, being the *BRAF*-mutated cases closer to healthy controls than *BRAF* wild-type counterpart. In agreement, a considerable score of correlation was also pointed out between bacteria abundance from *BRAF*-mutated cases and the level of markers distinctive of *BRAF*^V600E^ pathway, including those involved in inflammation, innate immune response and epithelial-mesenchymal transition. We provide evidence that two candidate bacterial markers, *Prevotella enoeca* and *Ruthenibacterium lactatiformans*, more abundant in *BRAF*^V600E^ and *BRAF* wild-type subjects respectively, emerged as single factors with the best performance in distinguishing *BRAF* status (AUROC = 0.72 and 0.74, respectively, 95% confidence interval). Furthermore, the combination of the 10 differentially represented microorganisms between the two groups improved performance in discriminating serrated CRC driven by *BRAF* mutation from *BRAF* wild-type CRC cases (AUROC = 0.85, 95% confidence interval, 0.69–1.01).

**Conclusion:**

Overall, our results suggest that *BRAF*^V600E^ mutation itself drives a distinctive gut microbiota signature and provide predictive CRC-associated bacterial biomarkers able to discriminate *BRAF* status in CRC patients and, thus, useful to devise non-invasive patient-selective diagnostic strategies and patient-tailored optimized therapies.

**Supplementary Information:**

The online version contains supplementary material available at 10.1186/s13046-020-01801-w.

## Background

Colorectal cancer (CRC) is the third most common cancer and the second leading cause of cancer-related deaths in developed countries [1]. Although some risk factors are well outlined [2] and many comprehensive studies have established the molecular criteria for CRC’s classification [1, 3, 4], the regulatory mechanisms of this tumor remain largely unrevealed.

The inherent extensive heterogeneity of CRC encompasses as many different histological and molecular bases associated with diverse clinical-pathological features. The adenoma-carcinoma sequence proposed by Vogelstein, in which the pre-neoplastic lesions accumulate stepwise molecular and morphological changes leading to cancer [5], has been long considered a unique model of CRC cancerogenesis until the description of the “serrated pathway” [6, 7]. This pathway arises from serrated polyps, once considered benign, including hyperplastic polyps (HPs), sessile serrated adenomas/polyps (SSAs/Ps) and traditional serrated adenomas (TSAs) [8, 9]. Among these, HPs are the most frequent (80–90%) and usually display a low malignant potential, while TSAs account for less than 1% of serrated CRCs [10]. SSAs/SSPs, which account for the 5–20% of all the serrated lesions, display peculiar molecular features, including CpG island methylator phenotype (CIMP-H) and *BRAF* mutation [9, 11]. CIMP-H results in transcriptional repression of *p16*^INK4a^ and *MLH1* genes, whereas *BRAF* mutations, often consisting in the activating V600E substitution, causes aberrant activation of the MAPK signaling [12, 13].

From a clinical point of view, *BRAF*-mutated CRC behave as a distinct subset compared to conventional adenomas, typically exhibiting lower response to conventional therapy, elevated invasiveness and the poorest clinical outcome, suggesting that initiation through this pathway might predict the aggressiveness of CRC [6, 13].

It is nowadays recognized that a small subpopulation of self-renewing CRC cancer stem cells (CCSCs) drive the initiation and progression of CRC, the metastatic colonization and the disease relapse after therapy [14, 15]. We have also very recently reported in our human CCSCs-based in vivo model that stemness underpins all the stages of CRC development, identifying CCSCs as a constitutive component in the establishment and dissemination of this tumor [16].

Disruption of the gut microbial community’s homeostasis, i.e. dysbiosis, also plays an important role in the initiation and maintenance of CRC as well as in the response to therapies [17–21]. In recent years a plethora of studies revealed the existence of a human CRC-specific microbial signature, underlining the pathogenetic role of some microorganisms, such as *Streptococcus gallolyticus*, *Bacteroides fragilis*, *Enterococcus faecalis*, *Fusobacterium spp*., *Fusobacterium nucleatum* and *Escherichia coli.* Yet, the expansion of these “bad” bacterial species with the ensuing depletion of “good microbes” has been shown to be implicated in DNA damage, uncontrolled cell growth, inflammatory signaling pathways and, thus, in CRC promotion and progression [22–24]. Additionally, it has been speculated that gut microbiota shift might be related to different early precursors of CRC, but whether specific microbial profiles could discriminate between conventional and *BRAF*-mutated CRC still remains under-investigated [25–27].

Here, we report either in our CCSCs-based in vivo model or in CRC patients that *BRAF*^V600E^ mutation can itself sustain a typical microbiota profile, thus identifying new putative tumor-associated bacterial markers for patient-tailored diagnostic and therapeutic purposes.

## Methods

Primary CCSCs culture and analysis, immunochemistry, reagents, targeted and sanger sequencing, qPCR and methylation-specific PCR, bacterial DNA extraction are described in detail in the Supplementary Methods.

### In vivo studies

All the animal experimental procedures were performed in compliance with the Guidelines for the Care and Use of Laboratory Animals. Animal protocols have been approved by the Ministry of Health (PR/15–297/2019-PR). In order to minimize any suffering of the animals, anesthesia and analgesics were used when appropriate. Orthotopical PDX was determined by injecting CCSCs into the wall submucosa of the ascending colon of *Scid/bg* mice (Charles River Lab) [16]. Quantification of tumor growth was performed from ventral and dorsal views by In vivo Lumina (Xenogen, PerkinElmer Inc) [16, 28]. Upon sacrifice at different time points according to the cell line originally injected, tissue samples from CCSCs-derived primary colon tumors and from mesenteric lymph nodes as well as lung, liver, spleen and brain metastases were collected and processed as previously reported [16, 28–30]. Spontaneous metastatic pulmonary lesions formation was performed by injecting 3 × 10^5^ luc-CCSCs cells into the lateral tail-vein of *Scid/bg* mice [16]. For the gut microbiota analysis, fresh fecal samples were collected from the cages at early-stage (7 days post-transplantation; DPT) and next to the median end-stage of the disease (late and end-stages, 37–43 DPT). Samples were immediately frozen and stored at − 80 °C until DNA extraction.

### Clinical patient’s features

Patients with a confirmed diagnosis of CRC (33 cases) and healthy subjects (13 subjects) included in the control group were enrolled in this study at IRCCS “Casa Sollievo della Sofferenza” Hospital, under the Ethical committee approvals number N.175/CE and N.94/CE. All the subjects agreed to participate according to the ethical guidelines of the 2013 Declaration of Helsinki and signed an informed consent. Eligible subjects were 45–90 years old who did not undergo radio/chemotherapy or pharmacological/long-term antibiotic treatments. All the fecal samples were collected at the moment of diagnosis before any surgery or adjuvant treatment. Human CRC tissues were classified according to established staging system (AJCC and TNM) and diagnosis was confirmed by the pathologist. Histological data together with localization of colonic lesions are reported in Table 1. Fresh stool samples were collected by each participant in containers with DNA stabilization buffer (Canvax Biotech) and stored at RT for few days until DNA extraction. Information regarding subject’s variables (i.e. age, gender and BMI) as well as dietary, lifestyle and smoking habits were assessed the same day of the stool sample collection.

**Table 1:** Characteristics of patients involved in the study

Sample name	Age	Sex	Location of cancer	AJCC Stage	*BRAF*^V600E^
MI4KC	63	M	Left colon	IIIA	wild type
MI6KC	62	M	Rectum	IIIB	wild type
MI7KC	73	M	Right colon	IIA	wild ttype
MI11KC	50	M	Rectum	IV	wild type
MI12KC	74	F	Left colon	IIIA	wild type
MI15KC	74	M	Right colon	IV	mutated
MI16KC	49	M	Rectum	IV	mutated
MI17KC	49	F	Left colon	IIA	wild type
MI23KC	53	F	Right colon	IV	mutated
MI22KC	67	M	Rectum	IV	mutated
MI27KC	80	F	Right colon	IIIA	wild type
MI31KC	81	M	Rectum	IIIB	wild type
MI32KC	80	M	Right colon	II	wild type
MI34KC	80	M	Right colon	IIA	mutated
MI36KC	51	F	Rectum	IIA	wild type
MI39KC	49	M	Rectum	IV	wild type
MI41KC	77	M	Right colon	IV	mutated
MI40KC	55	M	Left colon	IV	mutated
MI9KC	76	M	Rectum	I	wild type
MI10KC	87	F	Left colon	I	wild type
MI19KC	68	M	Right colon	I	wild type
MI20KC	85	F	Right colon	I	wild type
MI21KC	87	F	Left colon	I	wild type
MI25KC	70	M	Left colon	I	wild type
MI26KC	65	M	Rectum	I	wild type
MI35KC	72	F	Left colon	I	wild type
MI42KC	87	M	Right colon	I	wild type
MI43KC	67	M	Rectum	IV	mutated
MI38KC	59	F	Rectum	III	wild type
MI28KC	72	M	Rectum	IIA	wild type
MI14KC	59	F	Rectum	IIA	wild type
MI13KC	72	M	Right colon	IV	wild type
MI30KC	60	M	Right colon	I	wild type

### Sequencing and analysis of 16S rRNA

Library preparation and sequencing was performed with Illumina 16S Metagenomic Sequencing Library Preparation kit (Illumina Inc) accordingly to manufacture’s instruction. Briefly, the V3–V4 hypervariable region of the bacterial 16S ribosomal RNA was amplified with primers selected from [31], containing appropriate Illumina overhang adapter sequences. Amplicons were further amplified to attach dual Illumina indices (Nextera XT Index Kit, Illumina Inc) and PCR products again purified. The pooled libraries were paired-end sequenced (2 × 300 cycles) in Illumina MiSeq platform (Illumina Inc). Sequences were demultiplexed and FASTQ files were generated. Raw sequencing data were then trimmed for quality and Illumina adapters were removed. After excluding host reads, reads were aligned and mapped to the NCBI taxonomy database of bacterial and archaeal 16S rRNA sequences, using Kraken2 software [32]. Rarefaction curves were generated by randomly subsampling the OTU tables to a depth of 61,888 and 12,609 sequences (for mouse and human samples, respectively) per sample 10 times before computing the observed species. Several a-diversity metrics, including Chao1 and Shannon index, the Simpson reciprocal and the observed genus and species, were computed. To assess b-diversity in xenogeneic CRCs, jackknifed Bray–Curtis distances (10 sub-samplings at a depth of 61,888 sequences per sample) was computed and the matrices visualized in PCoA plot. Core diversity analysis was performed on the OTU tables, including a- and b-diversity as well as taxonomic summary, as implemented in QIIME software [33]. To account for library size, OTU profiles were converted to relative abundances and then filtered for species confidently detectable. Specifically, microbial species that did not exceed a maximum abundance of 1 × 10^− 3^ in at least one sample were excluded, together with the fraction of unmapped metagenomic reads. Hierarchical cluster analysis and visualization of the relative abundances were performed with Partek Genomics Suite v.6.6 software (Partek Inc.).

### Functional profile prediction

Based on the 16S rRNA sequences, microbial communities’ functional composition was predicted using PICRUSt software [34]. All sequences from each sample were searched against the Greengenes (gg_13_5) at the 97% identity (closed OTU picking method). OTU tables were normalized by dividing each OTU by the known/predicted 16S rRNA gene copy number abundance and the prediction of the metagenome functional content was classified according to the Kyoto Encyclopedia of Genes and Genomes (KEGG) Orthology. The predicted metagenome BIOM table was analyzed and visualized using the Statistical Analysis of Taxonomic and Functional Profiles (STAMP) v. 2.1.3 software [35].

### Statistical analyses

For in vitro studies, statistical tests were performed using GraphPad Prism v7.0 software and ANOVA tests according to the variance and distribution of data. Differential gene expression was assessed by the implementation of the ANOVA test available in Partek Genomic Suite 6.6 with FDR < 0.05. *P*-values< 0.05 were considered significant. Results from 16S rRNA gene sequences between or among groups were compared with nonparametric Mann-Whitney U, Student’s *t*-test (or Welch’s t-test for unequal variances) and Kruskal-Wallis test with Benjamini–Hochberg false discovery rate (FDR) correction for multiple comparisons at each level separately. FDR (*q*-value) < 0.10 was considered significant. Pearson correlation coefficient was used to assess association between gene expression and bacterial abundance at genus level in *BRAF*^V600E^ vs healthy and *BRAF* wt mice (43DPT) (*q*-values < 0.1). *P* < 0.05 was visualized. To discriminate the *BRAF* status in CRC patients, a Random Forest classifier (RF) was used [36]. The number of decision trees was set to 500. Significantly different bacterial species’ abundance between *BRAF*^V600E^ and *BRAF* wt CRC cases, as emerged from Mann-Whitney test (*P* < 0.05), was extracted as potential *BRAF* class biomarkers and selected as model features. From 8-fold cross-validation, mean test prediction scores were obtained and subjected to Receiver Operating characteristic (ROC) analysis. ROC curve was used to evaluate the diagnostic value of bacterial candidates in distinguishing *BRAF*-mutated from *BRAF* wt cases. A Fisher’s exact test was also performed. RF was firstly applied on the whole set of selected features and then on each one of them to identify the most important ones, ranked by areas under ROC (AUROC) metric. The best cut-off values were determined by ROC analyses that maximized the Youden index (J = Sensitivity + Specificity - 1 [31]. All statistical analyses were conducted using R software.

## Results

### Molecular and pathophysiological features of BRAF-mutated and BRAF wild-type CRC stem-like cells

**Fig. 1:**
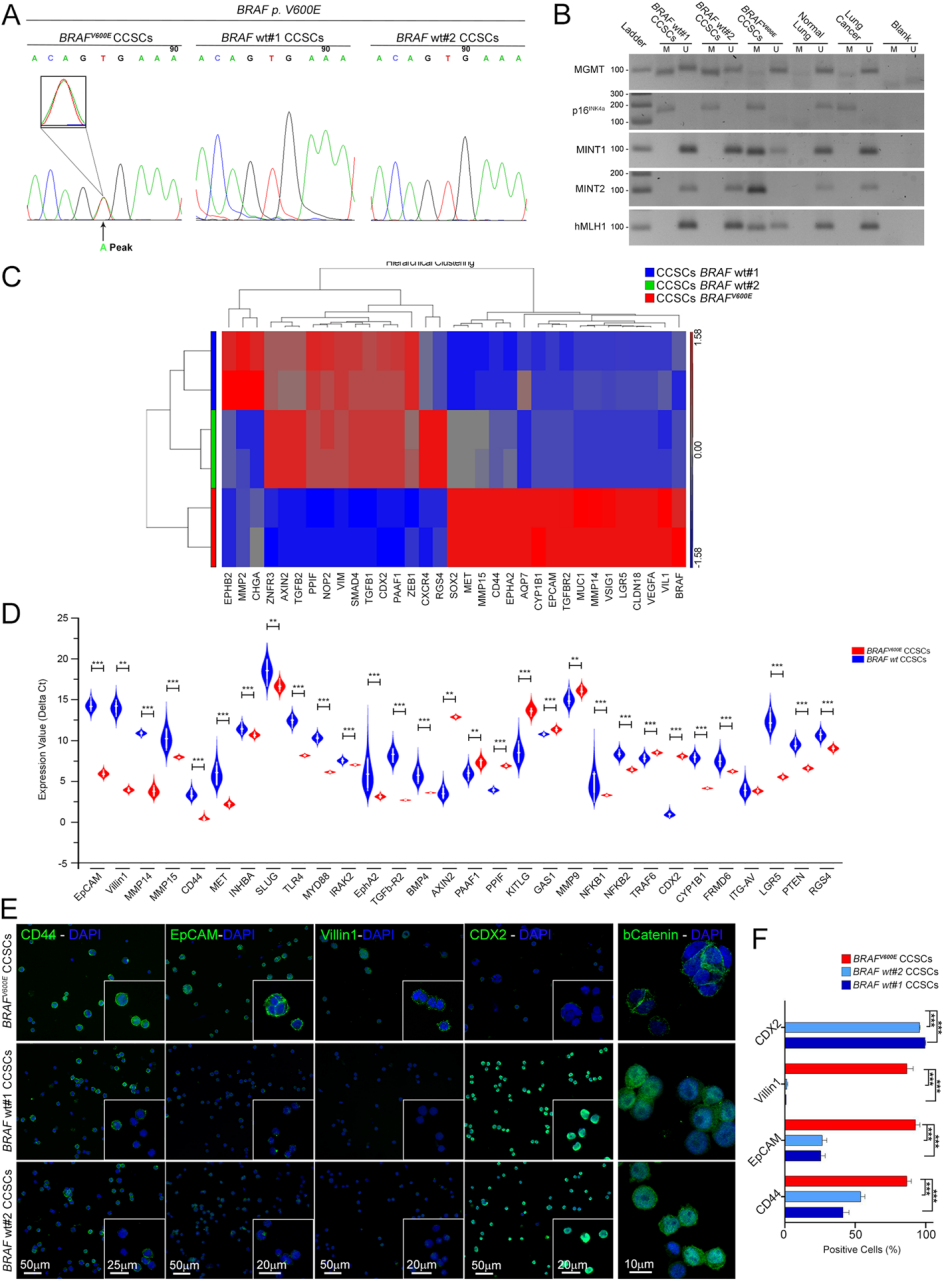
Phenotypic fingerprint of BRAF^V600E^ and BRAF wt CCSCs. **a** Automatic sequencing electrogram showing a minor “A” peak at T1796 denoting a CCSCs’ population retaining such a “T1796A” mutation with a T to A mutation in codon 599 (*BRAF*^V600E^ CCSCs; arrowhead). **b** *MGMT*, *p16*^INK4a^, *MINT1*, *MINT2* and *hMLH1* methylation was evaluated in *BRAF*^V600E^ and *BRAF* wt CCSCs by using primers for methylated (M) and unmethylated (U) alleles of bisulfite-treated DNA. Normal and cancer lung tissues as positive controls. **c** Heat map of one-way hierarchical clustering of 33 differentially expressed genes in *BRAF*-mutated vs. *BRAF* wt CCSCs revealing a typical CRC serrated signature for the former as compared to the latter. A dual-color code represents genes up- (red) and down-regulated (blue), respectively. **d** Differentially enriched genes associated with cellular migration and invasiveness, matrix degradation and epithelial phenotype in *BRAF*^V600E^ vs *BRAF* wt CCSCs, as confirmed by qPCR. ***P < 0.001, **P < 0.01, Mann-Whitney test. **e** By means of confocal imaging, widespread positivity for CD44, EpCAM and Villin1 markers and weak signal for CDX2 in *BRAF*^V600E^ CCSCs was shown. Positive nuclear b-catenin staining was retrieved exclusively in *BRAF* wt CCSCs. Insets: higher magnifications. Scale bars, 50um, 25um, 20um and 10um. Quantification of each marker is shown in **f**. ***P < 0.001, one-way ANOVA Tukey’s multiple comparison test. Data are mean ± SEM

By orthotopical injections of CRC stem-like cells (CCSCs) we have previously reported an in vivo model, which faithfully recapitulates human CRC features [16]. Here we characterized and confirmed in vitro and in vivo the phenotypic hallmarks of the three CCSCs lines isolated from CRC patient’s tissue, either associated to serrated pathway (*BRAF*^V600E^ CCSCs) or conventional CRC (*BRAF* wt CCSCs) [16]. To verify as to whether *BRAF*^V600E^ CCSCs reflects the key characteristics of serrated CRC, we delineated the presence of *BRAF* mutation and the unchanged form of *KRAS* and *NRAS* and the CIMP-H phenotype [37]. As shown in Fig. 1a, *BRAF*^V600E^ CCSCs are characterized by the presence of *BRAF* point mutation T1796A in exon 15 codon 599, reflecting a valine to a glutamic acid amino acid shift (V600E). Consistently, a methylated status of the promoter regions of *p16*^*INK4a*^*, MutL homolog1 (hMLH1), MGMT, MINT1* and *MINT2* genes was shown (Fig. 1b).

To pinpoint the inherent “serrated” signature of *BRAF*^V600E^ CCSCs, we next compared the transcriptional profile of CCSC lines to each other (Fig. 1c-e). As expected, hierarchical clustering analysis based on the global gene expression clearly segregated the serrated *BRAF*^V600E^ CCSCs from the other two CCSCs lines, which are quite similar (Fig. 1c). Consistently, among the genes preferentially expressed in *BRAF*^V600E^ CCSCs, many are reported to be up-regulated in the *BRAF*^V600E^ CRC pathway as well as to control matrix remodeling and epithelial-to-mesenchymal transition, inflammation and innate immunity, cell migration and invasion and transforming growth factor-b (Fig. 1d) [6, 38]. Meanwhile, transcriptomic fingerprint of *BRAF*^V600E^ CCSCs was identified by low levels of *CDX2* and *Wnt* target genes [39]. The epithelial cell adhesion molecule EpCAM, CD44 and VIL1 protein expression was confirmed to be a hallmark feature of CCSCs with a “serrated” phenotype, as compared to the *BRAF* wt counterpart, whereas CDX2 marker was highlighted exclusively in the latter (Fig. 1e-f). B-catenin protein was mainly localized in the plasma membrane in *BRAF*^V600E^ CCSCs cells (Fig. 1e) [16].

**Fig. 2:**
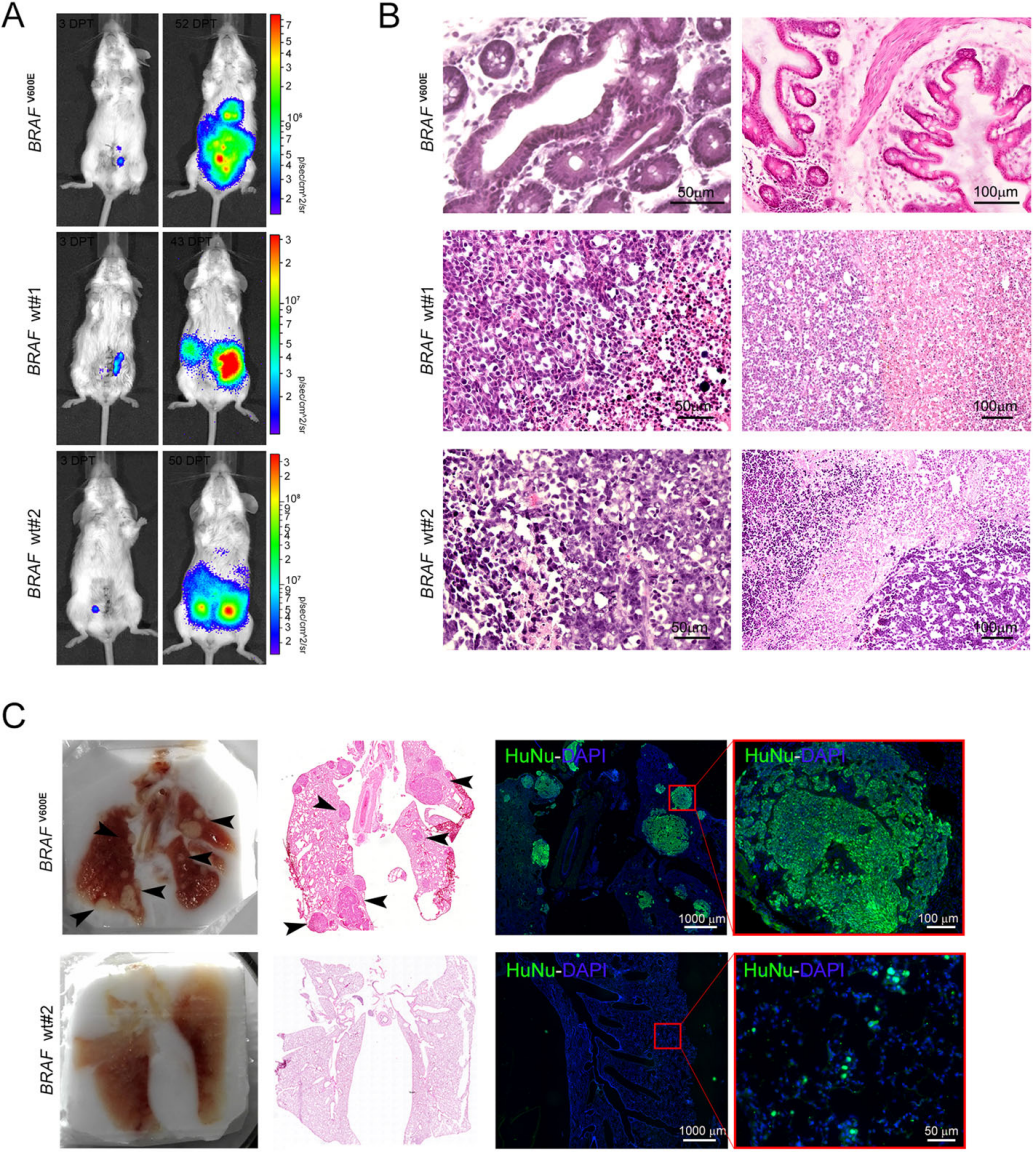
In vivo behavior of xenogeneic BRAF-mutated and BRAF wt CRC*.* **a** Quantitative time-course analysis of mice injected with luciferase-tagged *BRAF*^V600E^ (top) and *BRAF* wt CCSCs (middle and bottom). The progression of human CRCs was monitored from 3DPT (left) next to the end-stage disease typical of each CCSCs injected. **b** Histologic analysis, as expressed by H&E staining, revealing features of human serrated CRC with villiform architecture, micropapillary clusters and signet ring cells in *BRAF*^V600E^ lesions (top) while marked nuclear atypia and hemorrhagic necrosis (middle and bottom) were detected in *BRAF* wt CRCs. **c** OCT-embedded lung sections (left) marked with H&E (middle) depicting pulmonary lesions (arrowhead) in mice infused with *BRAF*^V600E^ CCSCs into the lateral tail-vein (*n* = 3 mice per groups). Widespread immunoreactivity for the human nuclei marker in *BRAF*^V600E^ lesions is shown (right). Scale bars, 1000um, 100um and 50um

Strikingly, following orthotopical delivery of *BRAF*^V600E^ CCSCs, tumors with histological architectures, that closely resemble the human serrated pathway, were detected, whereas the typical CRC morphology was identified in lesions from *BRAF* wt CCSCs-bearing mice (Fig. 2a-b). Though all CCSCs gave rise to distant spontaneous metastatic lesions [16], only mice infused intravenously with *BRAF*^V600E^ CCSCs exhibited pulmonary metastasis within 60 days after injection (Fig. 2c).

These findings lent to the conclusion that *BRAF*^V600E^ CCSCs recapitulates the main features of serrated CRC, being a faithful model for in vivo studies of serrated tumorigenesis.

### Microbiota profiles and functional composition of BRAF-mutated and BRAF wt xenogeneic CRCs

**Fig. 3:**
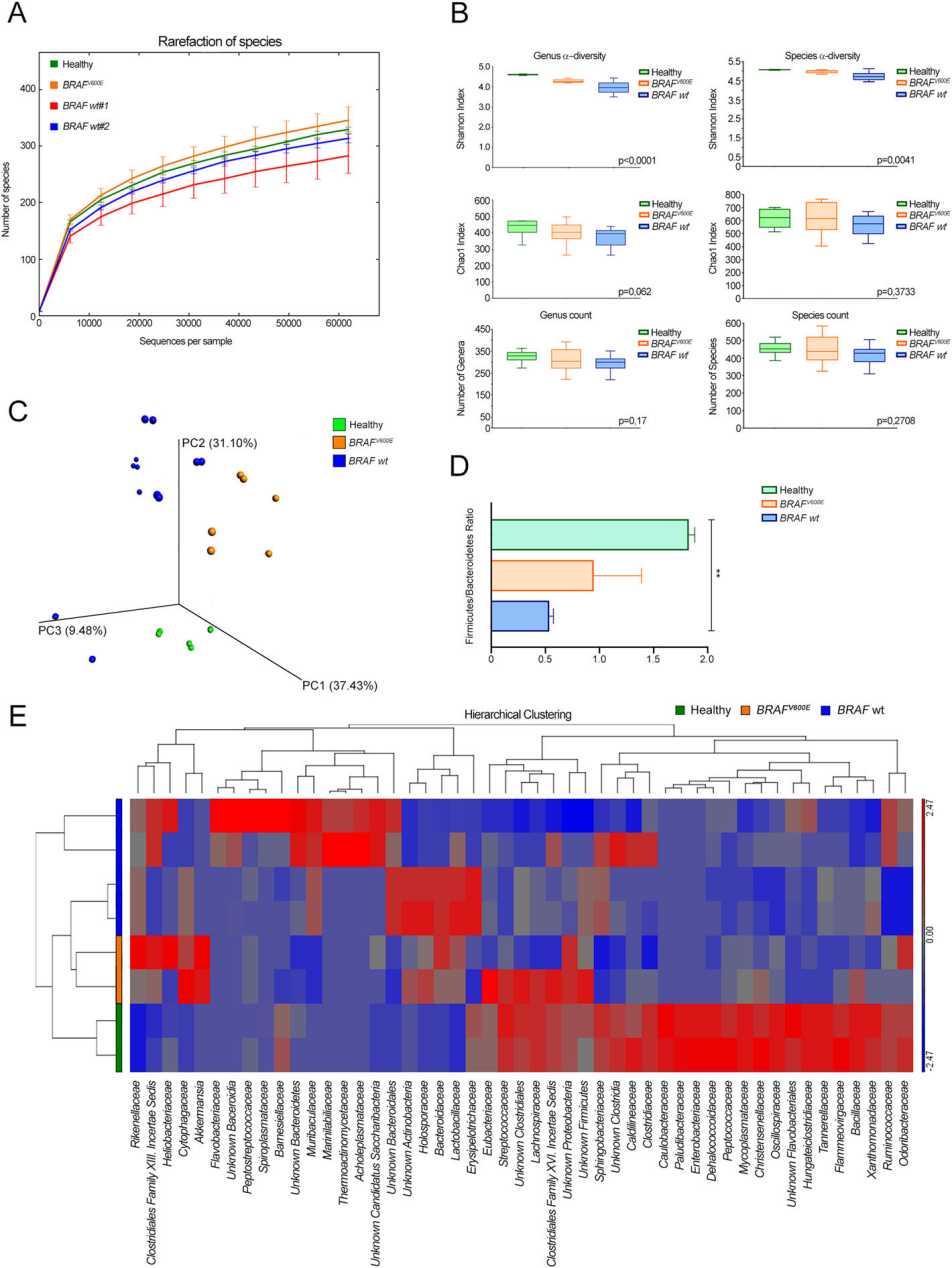
Microbial communities associated to BRAF^V600E^ and BRAF wt lesions. **a** Rarefaction curves for the analysis of microbial species content in the three groups of mice. **b** Microbial community a-diversity (top) and richness (middle and bottom) of the same groups at the genus (left) and species (right) level are shown. *P* values are from Kruskal-Wallis test are shown. **c** PCoA plot at the operational taxonomic unit (OTU) level showing the clustering pattern over time. **d** Ratio between *Firmicutes* and *Bacteroidetes* at 43DPT. **P < 0.01, one-way ANOVA Tukey’s multiple comparison test. Data are mean ± SEM. **e** Unsupervised hierarchical clustering analysis based on microbial communities’ presence at the family level showing an “healthy” profile in controls and a typical signature for each of the two CRC-carrier group. A dual-color code represents microbial families over-(red) and under-represented (blue)

**Fig. 4:**
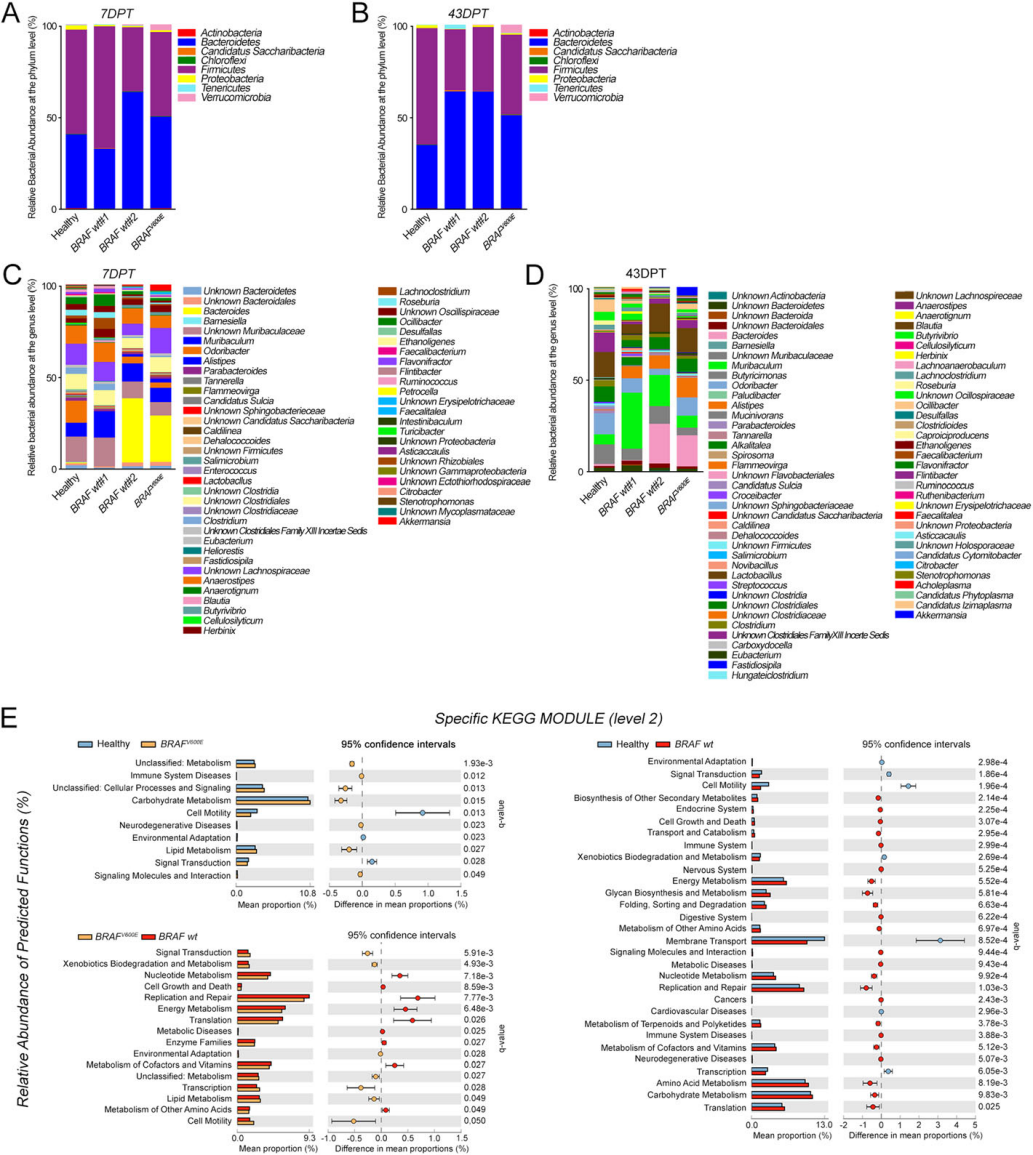
BRAF^V600E^ CRC exhibits specific bacterial markers and a typical functional microbiota composition. Gut microbiota relative abundance (% similarity) at the phylum (**a**-**b**) and genus level (**c**-**d**) in gut microbiota of mice carrying *BRAF*^V600E^ and *BRAF* wt CRC and controls at 7 DPT (**a**-**c**) and 43 DPT (**b**-**d**). **e** Significative relative abundance of predicted function (mean proportions %) and different abundance of predicted function (difference in mean proportion %) for specific KEGG modules level 2 in pairwise comparisons between controls, *BRAF*^V600E^ (left; top) and *BRAF* wt CRCs (right) or between the two CRC groups (left; bottom). FDR-adjusted *p* value *(q* value) from Mann-Whitney test are shown

To explore associations between gut microbiota composition and *BRAF*^V600E^ CRC, we first exploited our xenogeneic CRC model, in terms of global alteration in the microbiota profiles of *BRAF*^V600E^ and *BRAF* wt CRC-bearing mice vs control. Bacterial flora was analyzed either at early stage or next to the median end-stage of the disease (Figs. 3 and 4, Supplementary Table S1-S2). Species richness was found higher in controls and *BRAF*^*V600E*^ xenogeneic CRCs than in *BRAF* wt tumors (Fig. 3a). As indicated by the a-diversity Shannon index, xenogeneic *BRAF-*mutated CRC and controls displayed a higher microbial community diversity than *BRAF* wt CRC-carrier group, both at genus and species level (*P* < 0.0001 and *P* = 0.004, respectively, Kruskal-Wallis test) (Fig. 3b, top), whereas genus and species richness did not reach significance among the groups (Fig. 3b, middle and bottom). Next, we explored the signature of the gut microbiota in the CRC-carrier groups vs control observing in the former a remarkable clustering within the progression of the disease over time (Fig. 3c). *Firmicutes* and *Bacteroidetes* were found the most represented phyla in all groups, together with *Proteobacteria*, *Tenericutes* and *Verrucomicrobia* (Supplementary Table S1-S2). Yet, the key marker of gut dysbiosis [40] *Firmicutes/Bacteroidetes* ratio, tended to be comparable between xenogeneic *BRAF*^V600E^ CRCs and control, whereas was significantly lower in mice bearing *BRAF* wt tumors (Fig. 3d). Unsupervised hierarchical analysis at the family level (Fig. 3e) revealed a characteristic “healthy” microbial signature in controls segregated from that one of CRC-bearing mice, being *BRAF*^V600E^ CRCs closer to tumor-free mice than *BRAF* wt tumors. Notably, an enrichment in several butyrate-producing bacteria such as *Clostridiales*, *Eubacteriaceae*, *Lachnospiraceae, Proteobacteria, Firmicutes* and *Streptococcaceae* was observed in mice carrying *BRAF*^V600E^ CRC as well as in control.

Analyzing at the phylum level the bacterial flora of xenogeneic *BRAF*^V600E^ CRC versus control at 7 DPT (Fig. 4a and Supplementary Table S1), *Verrucomicrobia* (2.9% vs 0.2%, FDR = 0.005) and *Bacteroidetes* (47% vs 38%, FDR = 0.04) emerged more represented. Conversely, a significant lower presence of *Firmicutes* (43% vs 54%, FDR = 0.04), *Proteobacteria (*1% vs 2%, FDR = 0.04), *Tenericutes* (0.1% vs 0.2%, FDR = 0.04) and *Chloroflexi* (0.1% vs 0.3%, FDR = 0.04) was observed. Strikingly, these differences were abolished at 43 DPT (Fig. 4b and Supplementary Table S2). Yet, among the mostly represented bacterial genera in samples from *BRAF*^V600E^ CRC at 7 DPT (Fig. 4c and Supplementary Table S1), the highest abundance was found in *Bacteroides* (23.6% vs 0.5%, FDR = 0.05), *Akkermansia* (2.9% vs 0.3%, FDR = 0.007), *Butyrivibrio* (1.4% vs 0.3%, FDR = 0.004) and *Lactobacillus* (0.17% vs 0.07%, FDR = 0.03), whereas *Odoribacter* (2.6% vs 11.3%, FDR = 0.04), *Clostridum* (0.8% vs 3.1%, FDR = 0.009), *Roseburia* (0.5% vs 2.7%, FDR = 0.03) and *Ruminococcus* (0.4% vs 1.1%, FDR = 0.01) were among the most poorly represented. Conversely, at 43DPT all the identified genera were poorly present in *BRAF*^V600E^ CRC-bearing mice with respect to control (Fig. 4d and Supplementary Table S2). Consistently, all the differences detected down at the species level in *BRAF*^V600E^ CRC-carrying mice vs controls at 7 DPT were abolished at 43DPT (Supplementary Table S1-S2).

Concerning the shifts in the microbiota profile shared by the two *BRAF* wt CRC-carrier groups vs controls at 7 DPT, lower abundances of few genera either belonging to *Firmicutes* or to *Bacteroidetes* were detected (Fig. 4c and Supplementary Table S1)*.* Unlike xenogeneic *BRAF*^V600E^ CRC, at 43DPT, the relative bacterial abundance at the phylum and genus level seemed to be clearly different from that one of control (Fig. 4b, d and Supplementary Table S2). *Proteobacteria* (0.03 and 1% vs 1.5%, FDR = 0.035, FDR = 0.01) and *Bacteroidetes* (61.3 and 61.1% vs 33%, FDR = 0.03, FDR = 0.002) were shown to be phyla overrepresented in *BRAF* wt CRC-carrier groups, while *Firmicutes* were more abundant in controls (31.8 and 33.4% vs 60.1%, FDR = 0.035, FDR = 0.002). Further, consistently with Fig. 3e, *Roseburia* and *Lachnoanaerobaculum* genera, together with *Asticcacaulis* and *Ethanoligenens* declined in *BRAF* wt CRCs at 43 DPT, as compared to control.

When the microbial communities’ functional composition was compared between CRC-bearing mice and control (Fig. 4e and Supplementary Table S3), according to the level 2 KEGG module the relative abundance of immune system diseases (FDR = 0.012) category together with carbohydrate metabolism (FDR = 0.015) [41] emerged significantly higher in xenogeneic *BRAF*-mutated CRCs, whereas a remarkable enrichment of general metabolic functions was observed in *BRAF* wt CRCs. Consistently, when comparing the two CRC-carrier groups, cell motility (FDR = 0.05) and transcription (FDR = 0.03) categories were significantly enriched in *BRAF*^V600E^ CRCs, whereas metabolic functions were overrepresented in *BRAF* wt counterpart.

All of these data delineate a microbial signature associated to xenogeneic *BRAF*^V600E^ CRC, reminiscent of that one of control.

### Microbial taxa’s abundance correlates with gene expression in BRAF^V600E^ xenogeneic CRC

**Fig. 5:**
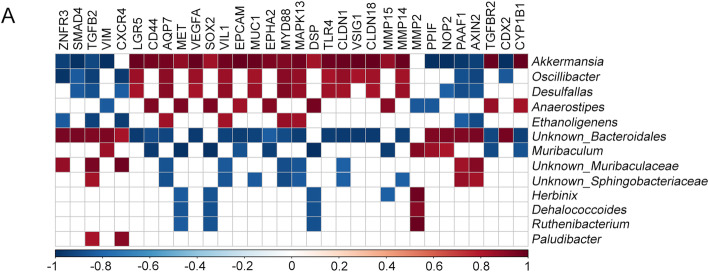
Correlation between BRAF^V600E^ CRC microbial composition and the level of serrated markers. **a** Pearson’s correlation coefficient computed between the relative abundance of microbial communities’ presence at the genus level in *BRAF*^V600E^ CRCs vs either the *BRAF* wt counterpart or control (*q* < 0.10) and the level of markers distinctive for serrated *BRAF*^V600E^ CCSCs. Significant correlation coefficients were visualized (*P* < 0.05). Red and blue, positive and negative correlation, respectively. Color intensity represents the increase/decrease of value

We then looked for a potential association between microbiota composition and the transcriptomic hallmarks of the *BRAF*^V600E^ serrated pathway [6]. For this purpose, the relative abundance of bacterial genera associated with xenogeneic *BRAF*^V600E^ CRC and the expression level of markers distinctive of *BRAF*^V600E^ CCSCs (Fig. 1e) were correlated with each other. A considerable score of positive correlation was identified between genera along with *Firmicutes* phylum, i.e. *Oscillibacter, Desulfallas, Anaerostipes* and *Ethanoligenens,* together with *Akkermansia,* and genes involved in the *BRAF*^V600E^ pathway, inflammation and innate immunity and epithelial-to-mesenchymal transition (Fig. 5a). Yet, a negative correlation trend was shown with almost all the *Wnt* target genes, *CDX2* and genes involved in the TGFb pathway*.* Remarkably, a specular correlation trend was shown for genera along with *Bacteroidetes* phylum, such as *Muribaculum* and genera belonging to *Bacteriodales*, *Muribaculaceae* and *Sphingobacteriaceae.*

Data here suggest the existence of a bidirectional communication, involving inflammation, invasion and innate immune signaling, between the *BRAF*^V600E^ lesion and the gut microbiota.

### Gut microbiota fingerprint in serrated BRAF^V600E^ and BRAF wt CRC patients

**Fig. 6:**
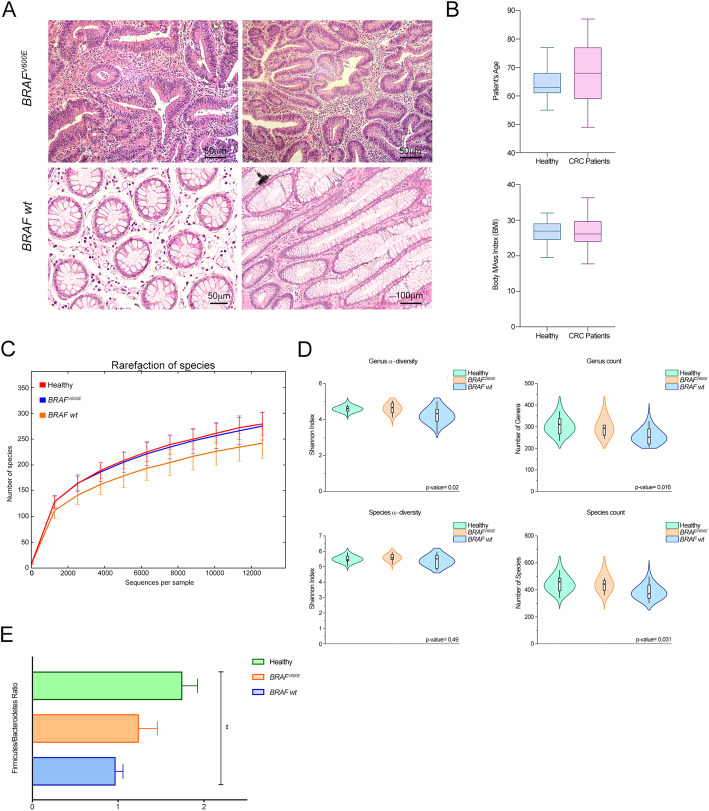
Microbial communities specific for BRAF-mutated and BRAF wt CRC patients. **a** Representative histologic analysis of patient’s tissues revealing a serrated architecture and the presence of dysplasia in *BRAF*^V600E^ CRCs (top) and traditional adenocarcinoma features in *BRAF* wt cases (bottom). Bar, 100um and 50um. **b** Age (top) and BMI (bottom) distribution in CRC and healthy subjects (*n* = 15 *BRAF* wt, 8 *BRAF*^V600E^ CRC, 13 healthy subjects), Mann-Whitney test. **c** Rarefaction curve for the analysis of microbial species content in healthy, *BRAF*^V600E^ and *BRAF* wt CRC cases. **d** A-diversity (Shannon index; left) and richness (right) of the three groups of subjects at the genus (top) and species (bottom) level. *P* values are from Kruskal-Wallis tests. **e** *Firmicutes* and *Bacteroidetes* ratio in the three groups of subjects. ** *P* < 0.01, one-way ANOVA Tukey’s multiple comparison test. Data are mean ± SEM

We next investigated the microbiota composition in a cohort of CRC patients who did not undergo any type of treatment (8 *BRAF*^V600E^ and 25 *BRAF* wt CRC) and healthy controls [13]. Early-stage stage I *BRAF* wt CRCs were excluded (Fig. 6a and Table 1) [41]. As shown in Fig. 6b, no significant differences were observed between CRC cases and controls in terms of age or the body mass index (BMI). Variants in *BRAF* were confirmed c. 1799 T > A mutation (p.V600E) (Table 1) and, as expected, a mutually exclusive missense mutation in *KRAS* and *NRAS* genes was found. When bacterial community properties were analyzed, the highest level of species richness was reported in *BRAF-*mutated and control groups vs *BRAF* wt cases (Fig. 6c). A-diversity and community richness were significantly different among the groups at genus level (*P* = 0.02 and *P* = 0.016, respectively, Kruskal-Wallis test) and, down at species level, displayed the lowest expression in *BRAF* wt cases (*P* = 0.031) (Fig. 6d).

**Fig. 7:**
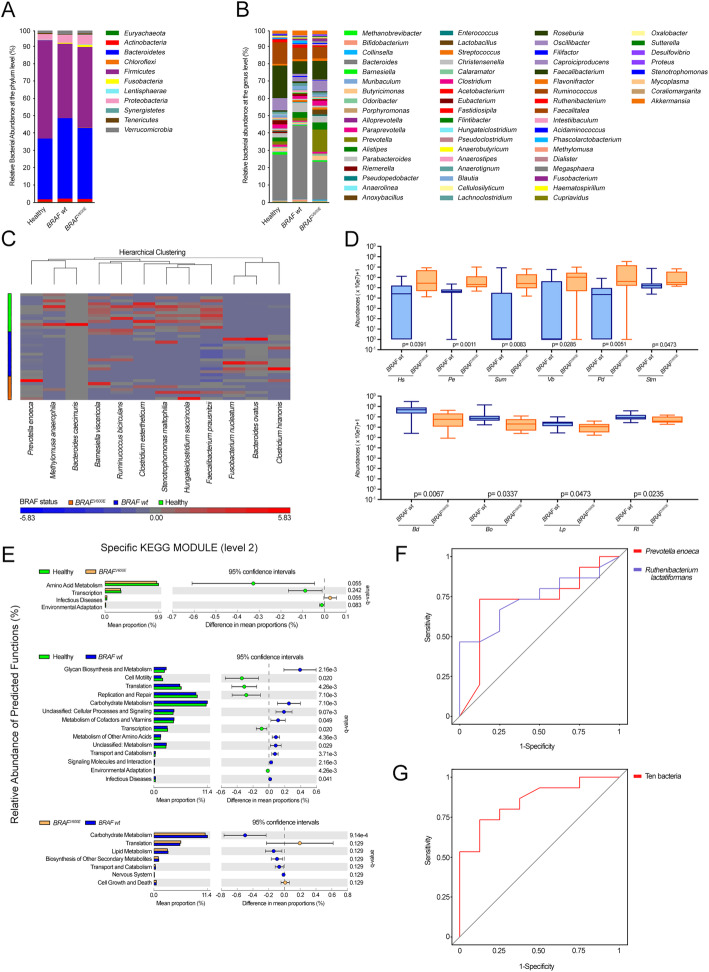
Identification of bacterial markers discriminating BRAF status in CRC patients. **a**-**b** Relative abundance of bacterial phyla (**a**) and genera (**b**) in fecal microbiota of healthy, *BRAF*^V600E^ and *BRAF* wt subjects. **c** Heatmap of one-way hierarchical clustering of differentially represented species among the three cohorts (*q*-values < 0.10 from Mann-Whitney test). A dual-color code counts for species up- (red) and down-represented (blue), respectively. **d** Differences in the relative abundances of *Hungateiclostridium saccincola* (*Hs*), *Prevotella enoeca* (*Pe*), *Sutterella megalospaeroides* (*Sum*), *Victivallales bacterium CCUG44730 (Vb), Prevotella dentalis* (*Pd*) and *Stenotrophomonas maltophila* (*Stm*) (top) and *Bacteroides dorei* (*Bd*), *Bacteroides ovatus* (*Bo*), *Lachnoclostridium phocaeense* (*Lp*) and *Ruthenibacterium lactatiformans* (*Rl*) (bottom) markers in *BRAF-*mutated vs. *BRAF* wt CRC counterpart. *P* values from Mann-Whitney test are shown. **e** Differences in the relative abundance of predicted function for specific KEGG modules (level 2) in pairwise comparisons between healthy and *BRAF*^V600E^ (top) or *BRAF* wt subjects (middle) and between *BRAF*^V600E^ and *BRAF* wt CRC cases (bottom). *q* values are from Mann-Whitney test. **f**-**g** Receiver operating characteristic (ROC) curves for the single metagenomic classifiers *Pe* or *Rl* (**f**) and for the combination of the 10 markers *Hs*, *Pe*, *Sum, Vb, Pd*, *Stm*, *Bd*, *Bo*, *Lp* and *Rl* (**g**) in discriminating *BRAF-*mutated from *BRAF* wt CRC cases

Strikingly, when comparing the gut microbiota’s signatures between CRC groups and healthy subjects, typical CRC-associated taxa emerged, being samples from *BRAF*^V600E^ patients closer to controls than *BRAF* wt (Fig. 7a-b and Supplementary Table S4). Significant differences among the three cohorts were found as for the relative abundance of *Firmicutes* phylum, highly represented in controls (54.6%) as compared to *BRAF*^V600E^ (45%) and *BRAF* wt (41.5%) cases (FDR = 0.01, Kruskal-Wallis test), and *Bacteroidetes,* whose abundance was higher in *BRAF* wt (45%) vs *BRAF*^V600E^ (40%) and controls subjects (34%) (FDR = 0.02). In line with Fig. 3d, the ratio between *Firmicutes* and *Bacteroidetes* phyla was comparable between *BRAF*^V600E^ and healthy subjects, while in *BRAF* wt cases was significantly lower (Fig. 6e). Yet, *BRAF*-mutated patients displayed the highest presence of *Fusobacteria* (1.2% vs 0.65 and 0.01%, *BRAF*^V600E^, *BRAF* wt and healthy, respectively; FDR = 0.01) and *Tenericutes* (0.61% vs 0.27 and 0.53%; FDR = 0.03). This finding was confirmed at the genus level (Fig. 7b and Supplementary Table S4). Yet, *BRAF*^V600E^ CRCs exhibited significant richness in *Fusobacterium* (1.2% vs 0.6 and 0.003%, *BRAF*^V600E^, *BRAF* wt and healthy, respectively; FDR = 0.05), reported to inhibit T cell-mediated immune response and to promote serrated carcinogenesis [27], and the lowest contribution in *Bacteroides* and *Proteus* (0.165% vs 0.002 and 0.016%; FDR = 0.03). Down at the species level (Fig. 7c and Supplementary Table S4), the highest presence of *Hungateiclostridium saccincola* was peculiar of *BRAF*-mutated samples (FDR = 0.04), while *Bacteroides ovatus* and *Clostridium hiranois* were characteristic of *BRAF* wt cases (FDR = 0.02 and FDR = 0.05).

Moreover, gut microbiota analysis in pairwise comparisons between healthy and CRC subjects, revealed that *Prevotella intermedia* (2.15% vs 0.005%) and *Sutterella megalosphaeroides* (0.13% vs 0.03%) were enriched in *BRAF-*mutated cases (FDR = 0.2 and FDR = 0.2, Mann-Whitney test), whereas higher abundance of *Clostridium hiranois* was retrieved in *BRAF* wt CRCs (0.4% vs 0.0004%; FDR = 0.01).

Yet, when *BRAF*^V600E^ and *BRAF* wt cases were compared to each other, two Bacteroides species, along with *Prevotella enoeca* (*Pe*) (0.158% vs 0.006%, *BRAF*^V600E^ vs *BRAF* wt, respectively) and *Prevotella dentalis* (*Pd*) (0.75% vs 0.01%), together with *Hungateiclostridium saccincola* (*Hs*) (0.2% vs 0.017%), *Sutterella megalosphaeroides* (*Sum*) (0.13% vs 0.07%), *Stenotrophomonas maltophilia* (*Stm*) (0.175% vs 0.07%) and *Victivallales bacterium CCUG44730 (Vb)* (0.22% vs 0.06%) emerged overrepresented in *BRAF*^V600E^ CRC, whereas *Bacteroides dorei* (*Bd*) (1.17% vs 7%, *BRAF*^V600E^ vs *BRAF* wt, *P* = 0.007, FDR = 0.2, Mann-Whitney test), *Bacteroides ovatus* (*Bo*) (0.4% vs 2.2%), *Ruthenibacterium lactatiformans* (*Rl*) (0.6% vs 1.25%, *P* = 0.02, FDR = 0.4) and *Lachnoclostridium phocaeense* (*Lp*) (0.1% vs 0.3%), were enriched in *BRAF* wt cases.

When the relative abundance of functional category of CRC cases was compared to healthy subjects, according to the level 2 and 3 KEGG modules, samples from *BRAF*^V600E^ CRC were confirmed to be closer to controls than *BRAF* wt CRC ones (Fig. 7e and Supplementary Table S5), perfectly matching data in Fig. 4e. When comparing the abundance of predictive function between *BRAF*^V600E^ and *BRAF* wt CRCs, translation (P = 0.02, FDR = 0.13) and cell growth and death (*P* = 0.03, FDR = 0.13) categories were significantly enriched in the former, whereas metabolic functions were overrepresented in the latter (Fig. 7e). Several functions of the genetic information processing category, including mismatch repair, ribosome and aminoacyl-tRNA biosynthesis were significantly highly expressed in *BRAF*^V600E^ vs *BRAF* wt cases, in which several functions of the metabolism, transport and catabolism categories were found, such as starch and sucrose, amino and nucleotide sugar metabolism and pentose phosphate pathway (Supplementary Table S5).

All of these data confirm that a distinctive microbiota’s fingerprint can be distinguished between serrated *BRAF*^V600E^ and *BRAF* wt CRC’s patients, with the former strongly resembling healthy subjects.

### Potential predictive biomarkers of BRAF status in CRC patients

We finally tested the predictive potential of the bacterial markers differentially represented in the two CRC groups to discriminate between *BRAF*-mutated and *BRAF* wt patients [22, 41, 42]. Among the 10 candidate species detected (Fig. 7d), *Rl* and *Pe* emerged as single factor with the best performance in discriminating *BRAF* status, as quantified by the area under the receiver operating characteristic (ROC) curve (AUC) of 0.74 and 0.72 (95% confidence interval, 0.53–0.95 and 0.51–0.93, respectively) (Fig. 7e). Performing ROC analysis at the best cut-off value that maximized the sum of sensitivity and specificity, *Pe* discriminated CRC patients based on their *BRAF* status with a sensitivity of 73%, specificity of 87.5%, negative predictive value (NPV) of 64% and positive predictive value (PPV) of 92% (*P*-value = 0.66, Fisher’s Exact Test, 95% confidence interval), while *Rl* showed a sensitivity of 47%, specificity of 100%, negative predictive value (NPV) of 50% and positive predictive value (PPV) of 100% (*P*-value = 0.62, Fisher’s Exact Test, 95% confidence interval). Strikingly, as depicted by the AUROC of 0.85 (95% confidence interval, 0.69–1.01) in Fig. 7f, the combination of all the 10 fecal markers reached a better performance in distinguish *BRAF*^V600E^ subjects, with a sensitivity of 73.3%, specificity of 87.5%, NPV of 63.6% and PPV of 91.7% (*P*-value = 0.026, Fisher’s Exact Test, 95% confidence interval).

Findings so far demonstrate that 10 candidate bacterial markers can discriminate *BRAF* status in CRC’s patients, representing new opportunities for the improvement of non-invasive identification and diagnosis of *BRAF*^V600E^ cases.

## Discussion

In this work, we provided the unprecedent findings that *BRAF*^*V600E*^ CRC subjects, who did not undergo any type of treatment, might be discriminated from the other CRC’s cases in terms of their microbial composition, being closer to healthy condition than *BRAF* wild-type cases. These findings were observed in *BRAF*^*V600E*^ CRC-bearing mice (Figs. 3 and 4 and Supplementary Table S2-S3), and, most important, confirmed in *BRAF*^*V600E*^ CRC patients (Figs. 6 and 7 and Supplementary Table S4-S5), pointing out that our in vivo model of serrated CRC recapitulates the main features of human disease.

We first analyzed gut microbial profile in terms of diversity and richness in CRC cases versus healthy controls and, in agreement with previous studies [43, 44], we found that *BRAF* wt cases, whether they were mice or patients, displayed lower a-diversity and richness (Fig. 3 and 6 a-b and c-d). Otherwise, these differences were not observed in *BRAF*-mutated subjects, which almost behave the same way as controls also in terms of *Firmicutes/Bacteroidetes* ratio, whose deviation is considered a hallmark of gut dysbiosis (Fig. 3 and 6d and e) [40]. Indeed, the down-representation of *Firmicutes* phylum observed in mice carrying *BRAF* wt CRCs, might also reflect the depletion of many “good” butyrate-producing bacterial families, such as *Clostridiales*, *Eubacteriaceae*, *Lachnospiraceae,* and *Streptococcaceae*, characterized by anti-inflammatory, immunoregulatory and metabolic functions [45, 46]. Importantly, as regards the molecular and functional microbiota composition over time, although numerous differences were detected at the beginning of the disease in both of the two groups of CRC-carrying mice versus control, only *BRAF*^*V600E*^ CRCs resembled healthy mice at the end-stage of the disease (Fig. 4 and Supplementary Table S2). Although the healthy status mirrored by *BRAF*^V600E^ is yet to be elucidated, our hypothesis is that the microbiota shift of *BRAF*-mutant CRC might be strongly related to the peculiar molecular profile of this tumor [6, 11, 38, 39]. Yet, one of the most relevant aspect emerging from the delineation of *BRAF*^V600E^ CRC’s microbiota profile, is likely found in the intriguing demonstration that several correlations do exist between bacterial genera abundance ad genes typically involved in the BRAF^V600E^ pathway (Fig. 5a). As expected, a positive trend of correlation for genes driving inflammation, innate immunity and invasion processes, mostly up-regulated in the *BRAF*-mutated pathway, was shown, whereas the typically down-regulated *CDX2* and *Wnt* target genes, displayed a negative trend [6, 11, 38, 39]. Furthermore, when comparing microbiota functional composition of *BRAF*-mutated versus wild-type counterpart a decrease in microbiota-associated metabolic functions was predicted (Figs. 4 and 7), thus confirming the capability of gut microbes to affect tumor through their metabolic functions, beside their impact on host immune and inflammatory responses [44]. These data not only suggested the existence of a bidirectional crosstalk whereby tumor impinge on gut microbiota and the microbiota influence tumor progression but also that a distinctive microbial fingerprint might be sustained by *BRAF*^V600E^ mutation itself. It is increasingly evident that microbial equilibrium plays a fundamental role in human health, since dysbiosis can contribute to or even initiate CRC development, through several mechanisms including triggering of a chronic inflammatory state, production of reactive oxygen species, genotoxins and carcinogenic compounds, interference with host immunity and metabolism [47, 48]. It should be noted, however, that the vast majority of investigation, addressed so far, concerned the role of gut microbiota in conventional CRC, whereas the few reports about the serrated-CRC mainly focused on the presence of *F. nucleatum*.

We demonstrated that, both in mice and in humans, BRAF-mutated CRC is characterized by a gut microbiota which, compared to conventional CRC, is more resembling to but still remains different from that of healthy subjects. This result was supported by previous findings by Peters et al., [26] in which conventional adenomas (precursor lesions of conventional CRC) but not SSAs (precursor lesions of the serrated pathway) were reported to display lower bacterial communities’ richness and diversity and a drop in butyrate-producing bacteria as compared to controls [26]. The reason why serrated BRAF mut CRC is associated to a more eubiotic condition is still to be clarified. One hypothesis is that gut dysbiosis may play a role only in the development of conventional CRC [25, 26], but not in the BRAF-mutated serrated one), which is also genetically, epigenetically, and molecularly different from the former. Nevertheless, we observed a positive correlation between bacterial genera found in BRAF mut microbiota and genes involved in inflammation, immunity and invasion processes typically expressed in the BRAF-mutated pathway. Another possible speculation is that, despite a microbiota composition generally resembling to that of healthy status, few or even single microorganisms (e.g. *F. nucleatum*) in the gut of BRAF mut CRC carriers might be sufficient to drive that specific carcinogenetic pathway.

Focusing on the microbiota composition of CRC patients enrolled in our study, an increased abundance of *Fusobacteria* in either *BRAF*-mutated or wild-type cases compared with controls emerged [27, 49]. Likewise, among the bacteria distinctive of each CRC group down at the species level, *Prevotella enoeca* and *Prevotella dentalis* were significantly enriched in patients harboring *BRAF*^*V600E*^ mutation (Fig. 7c-d), likely reflecting their massive presence in the biofilm lining the colonic mucosa [50]. Strikingly, as single factor, *Prevotella enoeca* together with *Ruthenibacterium lactatiforman,* overrepresented in *BRAF* wt cases, emerged as the species best discriminating *BRAF* status in CRC patients, therefore putative candidate non-invasive biomarkers (Fig. 7f). Furthermore, when considering the combination of all the 10 bacterial species differentially represented between the two CRC groups a best performance as a biomarker signature distinguishing *BRAF*^V600E^ from *BRAF* wt cases was reached, thus identifying potential diagnostic fecal biomarkers for CRC patients (Fig. 7g).

Thus, our work opens new and exciting possibilities for studying the biological underpinnings of the serrated *BRAF*^V600E^ CRCs and, most important, for the development of innovative diagnostic and therapeutic strategies for the cure of this deadly tumor.

## Conclusions

In the present study, we provide the unprecedented findings, observed in xenogeneic *BRAF*^V600E^ CRC and, most important, confirmed in patients harbouring *BRAF* mutation, that a distinctive microbiota profile could distinguish *BRAF*-mutated cases among CRCs. *BRAF*^V600E^ mutation drives itself a distinctive gut microbiota fingerprint in CRC, suggesting the existence of a bidirectional Tumor-Microbiota-Tumor connection. We identify a bacterial marker signature discriminating *BRAF* status in CRC patients, thus acting as reliable novel non-invasive clinical biomarkers for patient-tailored diagnostic and therapeutic applications.

## Supplementary Information

**Additional file 1:.** Supplementary methods

**Additional file 2: Supplementary Table S1.** Relative bacterial abundance at the phylum, genus and species level in fecal microbiota of mice at 7 DPT.

**Additional file 3: Supplementary Table S2.** Relative bacterial abundance at the phylum, genus and species level in fecal microbiota of mice at 43 DPT.

**Additional file 4: Supplementary Table S3.** Relative abundance of predicted function for specific KEGG modules (level 1–3) in fecal microbiota of mice at 43 DPT.

**Additional file 5: Supplementary Table S4.** Relative bacterial abundance at the phylum, genus and species level in fecal microbiota of human subjects.

**Additional file 6: Supplementary Table S5.** Relative abundance of predicted function for specific KEGG modules (level 1–3) in fecal microbiota of human subjects.

## Data Availability

Raw 16S rRNA sequencing data of all samples and raw Transcriptome array sequencing data were deposited in the Arrayexpress repository under accession code n. E-MTAB-9130 and n. E-MTAB-6940, respectively.

## Abbreviations

CRC: Colorectal carcinoma
CCSCs: Colorectal carcinoma stem-like cells
*BRAF* wt: BRAF wild-type
PDX: Patient derived xenograft
DPT: Days post transplantation
PCoA: Principle coordinates analysis
FDR: False discovery rate
KEGG: Kyoto encyclopedia of genes and genome
NPV: Negative predictive value
PPV: Positive predictive value
AUROC: Area under the receiver operating characteristic curve
*Pe*: *Prevotella enoeca*
Pd: *Prevotella dentalis*
*Hs*: *Hungateiclostridium saccincola*
*Sum*: *Sutterella megalosphaeroides*
*Stm*: *Stenotrophomonas maltophilia*
*Vb*: *Victivallales bacterium CCUG44730*
*Bd*: *Bacteroides dorei*
*Bo*: *Bacteroides ovatus*
*Rl*: *Ruthenibacterium lactatiformans*
*Lp*: *Lachnoclostridium phocaeense*
