# Supplementary material for: BRAFV600E mutation impinges on gut microbial markers defining novel biomarkers for serrated colorectal cancer effective therapies
Source: J Exp Clin Cancer Res. 2020 Dec 14;39:285. doi: 10.1186/s13046-020-01801-w (PMC7737386; doi:10.1186/s13046-020-01801-w)
Supplement: Supplementary file 1 — Additional file 1:. Supplementary methods [file 13046_2020_1801_MOESM1_ESM.pdf]

Supplementary Methods

Primary CCSCs culture and analysis. Stem-like colorectal cancer cells (CCSCs) were isolated from human CRC as previously described (1-3). Single-cell suspension was plated in NeuroCult NS-Amedium (Stemcell Technologies Inc) containing 20ng/ml of EGF (Peprotech Inc) and 10 ng/ml of FGF2 (Peprotech Inc) and cultured in humidified atmosphere at 37 °C, 5% O2 and 5% CO2 (1, 4, 5). CNV profiling was performed to authenticate the cell lines. Population, serial sub-clonogenic analysis and differentiation experiments were performed as in (1, 4, 5).

Immunohistochemistry and Immunocytochemistry. Hematoxylin and Eosin (H&E) staining were executed on OCT- and paraffin-embedded 10-um- and 4-um thick sections, respectively, and processed as previously described (1, 4). Images were acquired with Hamamatsu Scanner Nanozoomer SQA. For immunocytochemical analysis, cells were seeded onto Cultrex (Trevigen)-coated glass coverslips and stained with the appropriate antibodies (see following section), while cell nuclei were counterstained by DAPI (Roche Diagnostics) (1, 4-6). Images (n=5 fields/each sample) were analyzed by Nikon A1confocal and Zeiss Axioplan 2microscopes.

Antibodies and reagents. For immunofluorescence analysis antibodies/antisera used were: rabbit anti-EpCAM (Cell Signaling, 14452), rabbit anti-b Catenin (Cell Signaling, 8480), rabbit anti-CDX2 (AbCam, ab76541), mouse anti-Human Nuclei (Merck, MAB1281), mouse anti-CD44 (BD Biosciences, 550988), rabbit anti Villin 1 (Cell Signaling, 2369), goat anti mouse AlexaFluor488 (Thermo Fisher Scientific, A11001) and goat anti rabbit AlexaFluor488 (Thermo Fisher Scientific, A11008) secondary antibodies were used to visualize the primary antibody staining.

Sanger Sequencing. PCR for BRAF mutation V600E was performed as in (7) using the primers with the sequence:FW5'-TCATAATGCTTGCTCTGATAGGA-3' and RV 5'-GGCCAAAAATTTAATCAGTGGA-3'. PCR was performed using buffer condition of GoTaq DNA Polymerase (Promega, M7848), in 25 ul reaction mixture containing 20ng of gDNA. PCR products were separated and visualized using QIAxcel (an automated capillary electrophoresis system by Qiagen) and subsequently subjected to direct sequencing using the ABI Prism BigDye Terminator v3.1 Cycle Sequencing Kit (Applied

Biosystems). Sequences were determined using the automated AB3130xl (Applied Biosystems). Results were analyzed with Chromas Lite software (Technelysium) and Mutation Surveyor software (SoftGenetics).

Methylation-specific PCR (MSP). The methylation status of *p16*^{INK4a}, *hMLH1, MGMT, MINT1, MINT2* genes was analyzed in CCSCs cells by bisulfite treatment of DNA followed by MSP. 350 ng of genomic DNA was modified by sodium bisulfite treatment using an EZ DNA Methylation-Gold Kit (Zymo Research) and 2 ul of eluted DNA was used for PCR. The primer sequences and PCR methods were performed as in (7) and (8). PCR products from methylated and unmethylated reactions were electrophoresed on 2% agarose gel. DNA from normal and tumor lung tissues were used as negative and positive controls of methylation, respectively. PCR reaction without DNA was used as a negative control in each batch reaction. The methylation status of the same genes was analyzed in human CRC patient's samples extracted from FFPE. gDNA was subjected to bisulfite treatment and DNA purificated using the Epitect Bisulfite kit (Qiagen) according to manufacturer's instruction. Bisulfite-modified DNA was used as template for conventional MethylationSpecific PCR (MSP). PCR products were separated and visualized using QIAxcel (Qiagen).

Targeted Sequencing. DNA was extracted by using the QIAamp DNA Micro Kit (Qiagen) according to the manufacturer's instructions, from paraffin embedded blocks DNA quantity and quality were assessed by using the Qubit photometer (Thermo Fisher Scientific). 10 ng of dsDNA for each sample was used to prepare libraries with an in-house panel (Thermo Fisher Scientific) covering hotspot regions of 15 genes (*KRAS*, *NRAS*, *BRAF*, *EGFR*, *FGFR1*, *HRAS*, *PIK3CA*, *IDH1*, *IDH2*, *KIT*, *PDGFRA*, *CDKN2A*, *VHL*, *JAK2* and *TP53*). The quality of barcoded libraries was evaluated by High Sensitivity D1000 ScreenTape System on Agilent2200 TapeStation System (Agilent Technologies), before combining them to a final concentration of 100 pmoles. Template preparation by emulsion PCR (emPCR), enrichment of ion sphere particles (ISP), and loading on ION 510™ chip were performed by Ion Chef™ System with ION 510™/520™/530™ Kit – Chef (Thermo Fisher Scientific). Sequencing was carried out on Ion GeneStudio™ S5 System (Thermo Fisher Scientific).

The raw data were analyzed using the Torrent Suite Software v5.10 (Thermo Fisher Scientific). Mutations were detected using the Ion Reporter v5.12.0 software with high stringency settings and a 3% allelic frequency cut-off set to exclude false-positive results. Mutations were verified in the integrative genomics viewer (IGV) from the Broad Institute (http://www.broadinstitute.org/igv/), and their clinical relevance was evaluated by using ClinVar (https://www.ncbi.nlm.nih.gov/clinvar/) database.

Bacterial DNA extraction from human and mouse fecal samples. Human and mouse fecal DNA was isolated starting from 300µl of diluted (in stabilization buffer) stool sample and 200 mg of frozen feces respectively, using the QIAamp DNA Stool Mini Kit (Qiagen) according to the manufacturer's instructions to increase the ratio of microbial to host DNA. After the evaluation of concentration and purity, DNA was stored at -80° until processing.

Quantitative real-time analysis. Total RNA was extracted as in (6). cDNA was obtained by using SuperScript III Reverse Transcriptase (RT) (Invitrogen) and one ug of total RNA was primed with oligo-dT for cDNA synthesis. Quantitative PCR reactions were run in triplicate using IQ™ SYBR Green QPCR Supermix (Bio-Rad), fluorescent emission was recorded in real-time (Chromo 4 Four-Color Real-Time PCR Detector, Bio-Rad) and normalized to GAPDH as endogenous control. Gene expression profiling was completed using the comparative Ct method of relative quantification. The relative expression level is depicted in violin plot, that is, interquartile ranges (IQRs; thick bars), medians (open dots on the bars), the lowest and highest values within 1.5 times IQR from the first and third quartile (lines above and below the bars) and density of values (width between curves).

Supplementary References

1. Visioli A, Giani F, Trivieri N, Pracella R, Miccinilli E, Cariglia MG, et al. Stemness underpinning all steps of human colorectal cancer defines the core of effective therapeutic strategies. EBioMedicine. 2019;44:346-60.

2. Ricci-Vitiani L, Lombardi DG, Pilozzi E, Biffoni M, Todaro M, Peschle C, et al. Identification and expansion of human colon-cancer-initiating cells. Nature. 2007;445(7123):111-5.

3. Dalerba P, Clarke MF. Cancer Stem Cells and Tumor Metastasis: First Steps into Uncharted Territory. Cell Stem Cell. 2007;1(3):241-2.

4. Binda E, Visioli A, Giani F, Lamorte G, Copetti M, Pitter KL, et al. The EphA2 receptor drives self-renewal and tumorigenicity in stem-like tumor-propagating cells from human glioblastomas. Cancer Cell. 2012;22(6):765-80.

5. Galli R, Binda E, Orfanelli U, Cipelletti B, Gritti A, De Vitis S, et al. Isolation and characterization of tumorigenic, stem-like neural precursors from human glioblastoma. Cancer Res. 2004;64(19):7011-21.

6. Binda E, Visioli A, Giani F, Trivieri N, Palumbo O, Restelli S, et al. Wnt5a Drives an Invasive Phenotype in Human Glioblastoma Stem-like Cells. Cancer Res. 2017;77(4):996-1007.

7. JG H, JR G, S M, BD N, SB B. Methylation-specific PCR: A Novel PCR Assay for Methylation Status of CpG Islands. Proceedings of the National Academy of Sciences of the United States of America. 1996;93(18).

8. AO C, JP I, JS M, SR H, A R. Concordant CpG Island Methylation in Hyperplastic Polyposis. The American journal of pathology. 2002;160(2).
